# Evaluation of Methods and Processes for Robust Monitoring of SARS-CoV-2 in Wastewater

**DOI:** 10.1007/s12560-022-09533-0

**Published:** 2022-08-23

**Authors:** Olivera Maksimovic Carvalho Ferreira, Živa Lengar, Zala Kogej, Katarina Bačnik, Irena Bajde, Mojca Milavec, Anže Županič, Nataša Mehle, Denis Kutnjak, Maja Ravnikar, Ion Gutierrez-Aguirre

**Affiliations:** 1 National Institute of Biology, Večna pot 111, 1000 Ljubljana, Slovenia; 2 International Postgraduate School Jožef Stefan, Jamova cesta 39, 1000 Ljubljana, Slovenia; 3 School for Viticulture and Enology, University of Nova Gorica, Dvorec Lanthieri, Glavni trg 8, 5271 Vipava, Slovenia

**Keywords:** SARS-CoV-2, Method development, Detection, Wastewater, Monitoring

## Abstract

The SARS-CoV-2 pandemic has accelerated the development of virus concentration and molecular-based virus detection methods, monitoring systems and overall approach to epidemiology. Early into the pandemic, wastewater-based epidemiology started to be employed as a tool for tracking the virus transmission dynamics in a given area. The complexity of wastewater coupled with a lack of standardized methods led us to evaluate each step of the analysis individually and see which approach gave the most robust results for SARS-CoV-2 monitoring in wastewater. In this article, we present a step-by-step, retrospective view on the method development and implementation for the case of a pilot monitoring performed in Slovenia. We specifically address points regarding the thermal stability of the samples during storage, screening for the appropriate sample concentration and RNA extraction procedures and real-time PCR assay selection. Here, we show that the temperature and duration of the storage of the wastewater sample can have a varying impact on the detection depending on the structural form in which the SARS-CoV-2 target is present. We found that concentration and RNA extraction using Centricon filtration units coupled with Qiagen RNA extraction kit or direct RNA capture and extraction using semi-automated kit from Promega give the most optimal results out of the seven methods tested. Lastly, we confirm the use of N1 and N2 assays developed by the CDC (USA) as the best performing assays among four tested in combination with Fast Virus 1-mastermix. Data show a realistic overall process for method implementation as well as provide valuable information in regards to how different approaches in the analysis compare to one another under the specific conditions present in Slovenia during a pilot monitoring running from the beginning of the pandemic.

**Supplementary Information:**

The online version contains supplementary material available at 10.1007/s12560-022-09533-0.

## Introduction

The COVID-19 pandemic, caused by SARS-CoV-2, started in December 2019 in Wuhan, China. There are several modes of transmission including respiratory droplets, aerosols and direct contact with surfaces (Santarpia et al., 2020). Additionally, the virus is also shed through faeces and urine (Zhang et al., 2020), although, to date, faecal–oral route of transmission has not been confirmed (Sobsey et al., 2021). A meta-analysis showed that shedding of SARS-CoV-2 through faeces is present in 32–52% of symptomatic cases, and 15–44% of patients continue to shed the virus in the stool for additional 7 days after the loss of detectable viral RNA in their upper respiratory tract (Zhang et al., 2021a, 2021b). Reports indicate that asymptomatic individuals also shed the virus via the gastrointestinal tract (WHO, 2019, 2020). In addition to faeces, similar levels of shedding are also present in the urine of patients (Jones et al., 2020). In urban environments with well-developed communal infrastructure, most of the faeces and urine eventually enter the local sewage system and end up in wastewater treatment plants (WWTP). Monitoring of wastewater for the presence and the concentration of SARS-CoV-2 RNA has been shown to provide information about the scale of the epidemic in the population covered by specific WWTP. Wastewater-based epidemiology (WBE) has been successfully deployed in the past, e.g. for poliovirus outbreaks in Borno State in Nigeria (Deshpande et al., 2003). The first successful detections of SARS-CoV-2 in wastewater motivated a quick rollout of WBE in large cities (Medema et al., 2020). In parallel with the increase in the number of research groups working on WBE implementation, a variety of methods for collecting and processing the samples became available (Pecson et al., 2021). Currently, SARS-CoV-2 WBE studies vary with respect to nearly all steps of analysis, from sample type and storage through concentration and extraction method to final detection and quantification (Ahmed et al., 2022, Bivins et al., 2021). For sample storage, temperature and time are probably the most important factors that determine the stability of SARS-CoV-2 RNA in wastewater. Negative effects of ambient temperatures on the stability of the virus were reported relatively early (Ahmed et al., 2020a, 2020b), but the potential impact of prolonged cold storage and freeze/thaw cycles on the stability of SARS-CoV-2 in water samples also needs to be considered. Recent evidence in the literature suggests that freezing and thawing, as well as prolonged storage of the sample at − 20 °C, leads to a reduction in the measured RNA concentration of SAR-CoV-2 or surrogates (Alygizakis et al., 2021; Kaya et al., 2022; Steele et al., 2021). As viruses are generally present at low concentrations in environmental samples, such as wastewater, the selection of an appropriate concentration method is an important step to increase the sensitivity of detection. The main approaches typically used for concentration of viruses in water include: adsorption–extraction/elution, ultra-centrifugal filter devices, polyethylene glycol (PEG)-based precipitation and ultracentrifugation (Pulicharla et al., 2021). Nearly all of these methods were attempted or implemented in the WBE of SARS-CoV-2 (Jafferali et al., 2021; Kocamemi et al., 2020; Medema et al., 2020; Westhaus et al., 2021). For the nucleic acid extraction step, either automated magnetic methods (Kocamemi et al., 2020) or silica membrane spin columns approaches (Ahmed et al., 2020a, 2020b) are the most widely used approaches to purify the viral RNA from the concentrated wastewater samples. As most of these protocols have been developed for non-enveloped viruses, such as enteroviruses (Rusiñol et al., 2020), the protocols should be selected based on an evaluation in local laboratory conditions, as this will accommodate specific limitations introduced by the local wastewater and working conditions. The most widely used method for detection of SARS-CoV-2 in wastewater is real-time quantitative, reverse transcription PCR (RT-qPCR), with two or more assays employed simultaneously, targeting different parts of the same gene or different genes, to minimize the possibility of a false-negative or false-positive result (Zhang et al., 2021a, 2021b). To date an increasing number of different RT-qPCR assays targeting various regions of the virus, have been made available, including assays specifically designed to detect different variants of concern (Alygizakis et al., 2021; Bivins et al., 2021; Yaniv et al., 2021). In the literature there are increasing comparison studies and meta-analyses of all available RT-qPCR assays for detection of SARS-CoV-2 in wastewater, that have exposed the high variability between assays used for wastewater surveillance and call for harmonization efforts and adoption of quality checkpoints as, i.e. adoption of MIQE guidelines (Bivins et al., 2021; Zhu et al., 2021). The complexity of wastewater and its likely impact on the results indicate that any performance comparisons, before deployment of a WBE approach targeting SARS-CoV-2, should be done on real wastewater samples before making the final method choice. This and other important issues have been recently reviewed (Ahmed et al., 2022). On the global level, WHO has addressed these issues in the Interim Guidance document (WHO, 2022), which details points of consideration throughout the process of establishing WBE at a given location. Although it does not provide a recommended protocol, it does emphasize the need to evaluate and adjust to local conditions, which is conveniently described in several real case studies.

Our aim in this study was to describe the optimization steps that led up to the onset of a robust SARS-CoV-2 monitoring in wastewater in Slovenia. It includes an assessment of the influence of wastewater storage conditions in the SARS-CoV-2 detection by RT-qPCR; the applicability of different concentration protocols on real wastewater samples and a more detailed comparison of the best-performing ones; and finally the performance of different RT-qPCR assays in conjunction with different commercial mastermixes.

## Materials and Methods

### Thermal Stability Evaluation

To evaluate the impact of sample storage temperature and freeze–thaw cycles on the detection of SARS-CoV-2 RNA, we tested the stability of two different SARS-CoV-2 materials spiked in wastewater. Used materials included RNA from positive controls provided by European Virus Archive Global (EVA-GLOBAL) (concentration not calculated) and lyophilized, thermally inactivated SARS-CoV-2 virus propagated in cell culture provided by the Institut für Qualitätssicherung in der Virusdiagnostik, Berlin, Germany as part of INSTAND External Quality Assessment schemes (reconstituted following provider instructions to a concentration range of 4–11 × 10^9^ copies/mL, as reported by provider). These two materials were evaluated separately under the same experimental conditions by spiking either RNA from positive controls (EVA-GLOBAL) or the thermally inactivated virus into SARS-CoV-2 free composite wastewater collected in 2018 when SARS-CoV-2 was not present in Slovenia. Replicates with dilutions (1:10) of the spike materials in wastewater were stored at one of the selected temperatures/time sets that included + 4 °C, − 20 °C and − 80 °C, over a time span of 0 h to 7 days (see Supplementary Information; Table S1). For each temperature/time and spike material, the evaluation was done in three biological replicates. All extractions were done using QIAmp Viral RNA Mini Kit (Qiagen, USA, 52,906), following the manufacturer’s instructions with adjustments. Adjustments included double elution in 2 × 40 μL of nuclease-free water (Sigma-Aldrich, 3098) heated to 65 ℃ Each extraction batch was accompanied with at least one negative control of extraction (NCE) consisting of nuclease-free water added instead of the sample. Two ng of Luciferase Control RNA (Promega, USA, L4561) was spiked into each sample and NCE prior to extraction, to confirm the success of the extraction and to account for any inhibitory effects during the PCR reaction (data not shown). Viral RNA in the samples was determined by RT-qPCR with N1 and N2 assays as described in Sect. Evaluation of RT-qPCR Assay/Mastermix Combination. Cq values were plotted using the ggplot2 package in RStudio (v.1.2.1106) and visually inspected for noticeable trends.

### Sample Concentration Method Assessment

#### Concentration with Centricon 70-Plus Centrifugal Filters

Prior to processing any real wastewater samples, we performed a small evaluation trial on Centricon Plus-70 Centrifugal Filters with 10 kDa molecular weight cut-off (MWCO) (Millipore, Germany, UFC701008), the first method reported for detection of SARS-CoV-2 in wastewater (Medema et al., 2020). Two different spike materials (RNA from positive controls (EVA-GLOBAL) and thermally inactivated virus, described in Sect. Thermal Stability Evaluation) were used to confirm the performance of the filters. Each spike material was prepared as a 1:10 dilution in SARS-CoV-2 free composite wastewater influent sample and tap water. Concentration protocol was based on Medema et al., 2020, with some modifications. One hundred millilitres of the sample were centrifuged in two 50 mL Falcon tubes (Corning, USA 352,070) on 3200 × g for 50 min without break, using a swing-out rotor bucket (Eppendorf S-4–72) at room temperature. The supernatant was then filtered through the Centricon Plus-70 Centrifugal Filter unit in two consecutive rounds (50 mL + 50 mL) on 3200 × g with break and acceleration on ambient temperature, for 15 min or until the complete sample volume had passed through the filter (most often an additional 15 min cycle is enough). Collection of the concentrate was done by upside down centrifugation of the filter units on 1000 × g for 2 min, on ambient temperature. RNA from concentrated and non-concentrated fractions was extracted immediately using QIAmp Viral RNA Mini Kit (Qiagen, USA, 52,906) as described in Sect. Thermal Stability Evaluation and stored at −80 °C. Both the fraction collected before and the fraction collected after concentration, for each spike/matrix combination, were tested with RT-qPCR in triplicate using E assay (BHQ probe) as described in Sect. Evaluation of RT-qPCR Assay/Mastermix Combination. Additionally, they were tested with RT-qPCR assays for Luciferase Control RNA (Toplak et al., 2004), used as an RNA extraction and inhibition control (data not shown) and, for samples with wastewater as the matrix, also with pepper mild mottle virus (PMMoV) assay (Haramoto et al., 2013; Rački et al., 2014), to assess if this known faecal indicator, despite being so different structurally, is concentrated in the used setup with similar efficiency as the SARS-CoV-2, and therefore can be used as concentration efficiency control of the concentration procedure in SARS-CoV-2 surveys in wastewater. All assays were done in accordance with the description in Sect. General Technical Description for RT-qPCR, except the PMMoV assay which was performed in 2 technical replicates instead of 3. Concentration efficiency was evaluated based on the reduction in average Cq value before and after the concentration step.

To confirm the reproducibility of concentration with Centricon Plus-70 Centrifugal Filters 3 replicates from concentration to detection step of wastewater from WWTP Domžale-Kamnik were tested, using the same concentration and extraction protocols as described in the previous paragraph. Samples were then tested with RT-qPCR with N1 and N2 assays (Sect. Evaluation of RT-qPCR Assay/Mastermix Combination), Luciferase Control RNA (Toplak et al., 2004), (data not shown) and PMMoV assay (Haramoto et al., 2013; Rački et al., 2014). Standard deviation within both technical and all replicates was calculated with Excel 2010 built-in functions.

#### Evaluation of Additional Concentration Methods

Since the pandemic negatively affected the reliability of supply chains, in order to select a backup concentration procedure, we decided to evaluate other methods used for concentration of viruses from different types of water samples. A total of 13 samples from 7 different WWTP were included in the screening of concentration methods (Supplementary Information; Table S2 and Table S4). All WWTP samples were collected as a 24 h-flow-dependent composite sample of influent wastewater, volumes ranging from 200 mL to 2 L depending on the method (see Table 3). Samples were transported from the sampling point to the laboratory in cooling boxes. Samples were collected in November and December of 2020 and from January to April 2021. All samples were stored at 4 °C for a maximum of 48 h before processing. See Table 3 and Supplementary Information Table 4 for details on the processing of samples included in this experiment. Individual protocols included in the experiment are listed below and a schematic summary of each protocol is available in Supplementary Information Figure S1.

Centricon Plus-70 Centrifugal Filters with 10 kDa molecular weight cut-off (MWCO) (Millipore, Germany, UFC701008), were used to concentrate wastewater samples following the protocol described in 2.2.1. RNA from fractions collected before and fractions collected after concentration were extracted immediately using QIAmp Viral RNA Mini Kit (Qiagen, USA, 52,906) as described in Sect. Thermal Stability Evaluation and stored at − 80 °C.

Vivacell 100, 30,000 MWCO PES (Sartorius, Germany, VC1022) were used in an adapted version of the protocol used for Centricon Plus-70 Centrifugal Filters. 100 mL of the sample was centrifuged in two 50 mL Falcon tubes (Corning, USA, 352,070) on 3200 × g for 50 min without break, using a swing rotor bucket, on ambient temperature. The supernatant was pooled and then filtered through the Vivacell 100 unit on 3200 × g for 20 min, with break and acceleration on ambient temperature, or longer until the complete sample volume had passed through the filter. Fractions collected before and fractions collected after concentration were extracted immediately using QIAmp Viral RNA Mini Kit (Qiagen, USA, 52,906) as described in Sect. Thermal Stability Evaluation and stored at − 80 °C.

CIMmultus™ monolithic columns (BIA Separations, Slovenia) of various sizes (1 mL, 8 mL) and chemistries were evaluated using adapted protocols (Bačnik et al., 2020; Gutiérrez-Aguirre et al., 2011) (see Supplementary Information Table S3 for details listed in this paragraph). In each case, a different volume of wastewater sample (from 600 to 2000 mL) was pre-filtered through filter paper and cellulose acetate filter membrane with a pore size of 0.8 μm (Sartorius, Germany, 11,104–142) and loaded onto the corresponding preconditioned column (following manufacturer’s recommendations) using fast protein liquid chromatography system AKTA Purifier 100 (GE Healthcare, USA). The flow rate was adjusted to keep the backpressure stable at a fixed limit. After a wash step using 20 × column volumes, an elution step was performed with different volumes (8 mL to 20 mL) of a high ionic strength elution buffer. The elution peak was monitored by measuring UV absorption at 280 nm and conductivity. In between samples, columns were sanitized with 1 M NaOH for 120 min. Collected fractions included: sample before filtration—raw (R), sample after filtration—load (L), flow-through of the sample through the column (FT), wash step (W) and elution (E), all of which were extracted immediately using QIAmp Viral RNA Mini Kit (Qiagen, USA, 52,906) as described Sect. Thermal Stability Evaluation and stored at − 80 °C.

Concentration using polyethylene glycol (PEG) was performed using a protocol made publicly available by IDEXX Laboratories (IDEXX, 2020) without a pasteurization step. Fractions collected before and fractions collected after concentration were extracted immediately using QIAmp Viral RNA Mini Kit (Qiagen USA, 52,906) as described in Sect. Thermal Stability Evaluation and stored at − 80 ℃.

Concentration using skimmed milk flocculation was based on the protocol described in Calgua et al., 2008, with adaptations. Sample (200 mL) was left to stir at room temperature for 6 h and then centrifuged at 3200 × g for 30 min on ambient temperature. The supernatant was carefully discarded and the pellet suspended in 800 µL of phosphate buffer. Fractions collected before and fractions collected after concentration were extracted immediately using QIAmp Viral RNA Mini Kit (Qiagen, USA, 52,906), as described in Sect. Thermal Stability Evaluation and stored at − 80 °C.

For direct RNA capture, Wizard Enviro TNA Kit (PROMEGA, USA, A2991) and Maxwell RSC Enviro TNA Kit (PROMEGA, USA, AS1831) were used according to manufacturer’s instructions. Both kits already include an RNA extraction step (Wizard Enviro TNA Kit relies on silica spin columns and Maxwell RSC Enviro TNA Kit on semi-automated magnetic beads system);

Each extraction batch was accompanied by at least one negative control of extraction (NCE) consisting of nuclease-free water added instead of the sample. Also, in order to evaluate extraction efficacy and potential inhibitory effects, each sample and the NCE were spiked with 2 ng of Luciferase Control RNA (Promega, USA, L4561) at the beginning of extraction. Extracted RNA was stored on −80 °C until further analysis.

RNA extracts from concentrated and non-concentrated fractions, for each described protocol, were tested with RT-qPCR (N1 and N2 assays). Additionally, they were tested with RT-qPCR assays for PMMoV (Haramoto et al., 2013; Rački et al., 2014), which served as an additional indicator of each method’s concentration efficiency, and Luciferase Control RNA (Toplak et al., 2004), used as an RNA extraction and inhibition control. Similar Cq values obtained for luciferase in all analysed samples excluded the presence of inhibition. RT-qPCR reactions were performed as described in Sects. Evaluation of RT-qPCR Assay/Mastermix Combination and General Technical Description for RT-qPCR. Concentration efficiencies for different approaches were evaluated based on the reduction in average Cq value before and after the concentration step.

Both in the screening for concentration methods as well as in the comparison among Centricon Plus-70 and Wizard enviro TNA kit, we report Cq value reductions after concentration by each method of the same given water sample. The starting and concentrated volumes are shown in Table 3. Recoveries were not calculated, and they could differ due to different start and end volumes for each method. However, our aim was to select the method resulting in the lowest Cq value after the concentration of the same sample, regardless of the recovery, as such method would result in the highest sensitivity of the RT-qPCR quantification.

### Evaluation of RT-qPCR Assay/Mastermix Combination

In order to select an assay, which would allow sensitive detection and accurate quantification of SARS-CoV-2 RNA in wastewater, we tested 4 primer/probe sets (assays) available in the early stages of the pandemic for detection of SARS-CoV-2 genome: RdRp (Corman et al., 2020), E (Corman et al., 2020) with two different quenchers, N1 (CDC, USA, 2020) and N2 assays (CDC, USA, 2020). Details regarding primer and probe sequences with quencher information are available in Supplementary Information, Table S5.

Evaluation of the performance of all RT-qPCR assays was done on dilution series of thermally inactivated SARS-CoV-2 virus (described in Sect. Thermal Stability Evaluation). The reconstituted virus was used as a starting point for serial dilutions (scheme in Supplementary Information; Table S6). Dilutions were done in a 24h composite influent wastewater sample that had previously tested negative for the presence of SARS-CoV-2. RNA was extracted from each dilution using QIAmp Viral RNA Mini Kit as described in Sect. Thermal Stability Evaluation RT-qPCR analysis of serial dilutions was done in triplicates for all 5 SARS-CoV-2 target assays using TaqMan Fast Virus 1-Step Master Mix (Thermo Fisher Scientific, USA, 4,444,432). Assays were compared to one another based on the standard deviation between the three replicates in the lowest detected dilution (data not shown).

The second phase of the performance evaluation included the assessment of three commercial mastermixes. As reagent shortages were expected it was important to understand how the change of mastermix could impact the results. Dilutions of thermally inactivated SARS-CoV-2 virus in wastewater, described in the previous section, were tested using N1 and N2 RT-qPCR assays using the following mastermix kits, all used according to manufacturer’s recommendations: TaqMan Fast Virus 1-Step Master Mix (Thermo Fisher Scientific, USA, 4,444,432), RNA UltraSense™ One-Step Quantitative RT-PCR System (Thermo Fisher Scientific, USA, 11,732,927) and AgPath-ID™ One-Step RT-PCR Reagents (Applied Biosystems, USA, AM1005). The assessment was done in two independent serial dilutions with different dilution steps (Supplementary Information; Tables S6 and S7). For each assay/mastermix the limit of quantification (LoQ) was defined as the concentration of the highest dilution at which the variance coefficient of measured Cq values between three technical replicates was below 0.5, and the limit of detection (LoD) was set to be the concentration of the dilution, at which at least one out of three technical replicates were positive. When a replicate measurement was clearly different from the other two replicates within the triplicate, it was not considered in the calculations. The selection of the mastermix was based on the sensitivity and accuracy of detection on the highest dilution.

### General Technical Description for RT-qPCR

Each RT-qPCR reaction was performed with 2 µL of extracted RNA per reaction in a total reaction volume of 10 µL. RT-qPCR analysis was done using Applied Biosystems™ 7900 HT Fast PCR Instrument (Thermo Fisher Scientific, USA, 4,329,001) with cycling parameters as recommended by the individual mastermix manufacturer. Data were analysed using SDS 2.4.2 Standalone software with automatic setting of the baseline and threshold for RdRp and E assays and manual threshold set up for N1 (0.11) and N2 (0.12) assays. Each amplification plot was checked manually and the result was considered positive if it produced an exponential amplification curve distinguishable from negative controls, and in such cases, Cq values were calculated. Every RT-qPCR plate included positive controls and a no template control. Positive controls were synthetic single-stranded RNA (ssRNA) fragments of SARS-CoV-2 (EC-JRC, EURM-019), 1.000x (expected Cq 21) and 100.000x (expected Cq 28) diluted in nuclease-free water. Results from positive controls were monitored within a control chart, which indicates if the value was within a predefined range (± 3Cqs, data not shown).

## Results

### Thermal Stability Evaluation

**Fig. 1:**
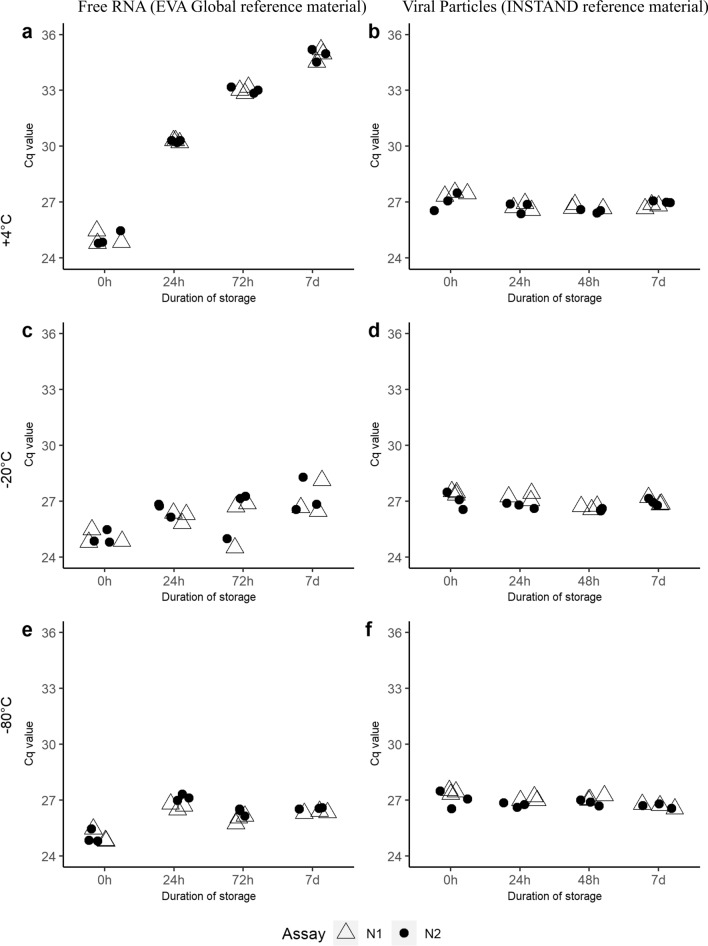
Overview of stability evaluation for wastewater spiked with RNA from positive controls (EVA-GLOBAL) stored for different time periods on + 4 °C (**a**); − 20 °C (**c**) and − 80 °C (**e**) and wastewater spiked with the thermally inactivated virus for different time periods on same temperatures of + 4 °C (**b**); − 20 °C (**d**) and − 80 °C (**f**). Each incubation was repeated 3 times in parallel and the points show average Cq values from RT-qPCR (*n* = 3) for both N1 and N2 assays. Figure created in RStudio v.1.2.1106

The RT-qPCR detection (Cq values obtained with both N1 and N2 assays) of thermally inactivated virus spiked in wastewater remained stable at each temperature/time (Fig. 1b, d and f). For spiked SARS-CoV-2 RNA from positive controls (EVA-GLOBAL) we observed rapid degradation in wastewater, especially at 4 °C, where the Cq values increased over 6 Cq values already after 24 h (Fig. 1a), but also at − 20 °C and − 80 °C, where we observed an increase of 2 to 3 Cq values. The observed increase in Cq values remained on average similar in all tested storage times (Fig. 1c, e).

### Sample Concentration Method Assessment

#### Centricon 70-Plus Centrifugal Filters

In the first experiment with Centricon Plus-70 Centrifugal Filters both synthetic RNA and thermally inactivated virus were concentrated similarly from 100 mL of spiked tap water to 1 mL, with an average Cq reduction of 5.1 (synthetic RNA) and 5.6 (thermally inactivated virus) (Table 1). In ideal conditions (100% efficiency of the concentration method) the viral concentration should have increased by 100-fold, meaning a Cq decrease of 6.68 (at 100% amplification efficiency of RT-qPCR). The observed Cq reductions thus confirm that Centricon 70 Plus is a suitable tool for concentration of both naked RNA and packed viral particles. Synthetic RNA spike showed different behaviour in tap water and wastewater. Looking at the Cq values before the concentration step, we can see an increase in values in the wastewater, likely as a consequence of faster degradation in comparison to tap water, which seemed to happen at a larger extent with RNA from positive controls (EVA-GLOBAL) (previous section). This is confirmed by the results with synthetic RNA after concentration in wastewater, by that time we already observed a complete loss of a detection signal, which was not due to the inhibition of PCR reaction as confirmed by the lack of inhibition in the same sample spiked with viral particles (Table 1) and also from Cq values obtained for Luciferase Control RNA (internal amplification control) assay, which were comparable in all samples (data not shown). Both, the spiked thermally inactivated virus and the naturally occurring PMMoV, which has been proposed to be used as a measure of the faecal contribution in WBE analysis (Kitajima et al., 2018), were concentrated similarly in wastewater samples with Cq reductions of 4.9 (synthetic RNA) and 5.2 (thermally inactivated virus) (Table 1).

**Table 1:** Cq values and reduction of average Cq values obtained by concentration with Centricon Plus-70 Centrifugal Filters of tap water and wastewater spiked with either synthetic RNA or thermally inactivated virus

Water type	Tap water		Wastewater		Wastewater	
Target	SARS-CoV-2; E-gene		SARS-CoV-2; E-gene		PMMoV	
Spiked with	Synthetic ssRNA	Thermally inactivated virus	Synthetic ssRNA	Thermally inactivated virus	Synthetic ssRNA	Thermally inactivated virus
Before concentration (Cq)	22.4	27.5	27.1	28.0	26.0	26.4
	22.7	27.5	27.2	28.0	25.9	26.1
	22.6	27.8	27.6	27.9	–	–
After concentration (Cq)	17.7	22.1	Undetected	23.1	20.1	21.1
	17.7	21.9	Undetected	23.0	20.2	21.0
	16.8	21.9	Undetected	23.1	–	–
Average Cq reduction	5.1	5.6	N/A	4.9	5.8	5.2

Cq values presented for E assay (BHQ quencher) and PMMoV

The procedure (from concentration step to detection step) showed good reproducibility in concentrating SARS-CoV-2 and PMMoV from a real wastewater influent sample based on the low intra- and inter- replicate standard deviation of Cq values obtained in three concentration rounds (Table 2).

**Table 2:** Cq values (shown in triplicate for N1, N2 and PMMoV assays) obtained for each of the three independent replicate concentration/detection rounds done in the same wastewater influent sample

Replicate/Target	Cq (SARS-CoV-2; N1)	Intra-replicate standard deviation	Cq (SARS-CoV-2; N2)	Intra-replicate standard deviation	Cq (PMMoV)	Intra-replicate standard deviation
Replicate 1	31.3		31.7		21.9	
	32.3	0.4	31.3	0.2	21.4	0.4
	31.8		31.5		21.1	
Replicate 2	30.9		31.3		21.3	
	31.1	0.1	31.7	0.2	20.8	0.3
	31.2		31.3		20.6	
Replicate 3	31.7		31.5		20.8	
	31.4	0.4	31.2	0.1	20.8	0.0
	30.7		31.4		20.8	
Inter-replicate standard deviation among all replicates	0.5		0.2		0.4	

Intra- and inter-replicate standard deviations are also shown

#### Evaluation of Alternative Concentration Methods

When looking at the range of tested concentration protocols, with most of them we were able to concentrate naturally occurring PMMoV (Cq reduction ranging from 2.5 to 6.0), except for the positively charged CIMmultus™ monolithic columns (BIA Separations) with SO3 chemistry (Table 3). In most tested protocols we were concentrating SARS-CoV-2 less efficiently than PMMoV. Skimmed milk-based protocol and Vivacell 100, 30,000 MWCO PES (Sartorius, Germany, VC1022) resulted in almost no Cq reductions for N1 and N2 assays (Table 3). Along with Centricon Plus-70 Centrifugal Filters, the Wizard Enviro TNA Kit (PROMEGA, USA, A2991) outperformed all other methods for simultaneous concentration of both targets, with Cq reductions ranging from 4.9 to 6.9 for SARS-CoV-2 and Cq reduction of 4.3 to 6.0 for PMMoV (Table 3) on starting volumes of 40–100 mL. In terms of price per sample, all methods except ones relaying on CIMmultus™ monolithic columns (BIA Separations) have acceptable price points below 300€ per sample. Similarly, the time required to process a batch is within the timeframe of one working day. However looking at the number of samples that can be processed simultaneously within one batch Wizard Enviro TNA Kit (PROMEGA, USA, A2991) provides the best platform for high throughput analysis as it can accommodate the highest number of samples per batch.

**Table 3:** Concentrations achieved by different methods from the same wastewater sample expressed as a reduction in average Cq value, from RT-qPCR replicates (marked as ΔCq; *n* = 3) before and after concentration for each performed assay (N1, N2, PMMoV)

Concentration method	Start volume (mL)	End volume (mL)	ΔCq N1	ΔCq N2	ΔCq PMMoV	Samples per batch	Price per sample	Time per batch
Centricon Plus-70 Centrifugal Filter *Experiment 1*	100	0.5	5.1	5.9	5.7	8	245 €	7 h
Centricon Plus-70 Centrifugal Filter *Experiment 2*	100	0.5	6.2	5.7	4.3			
Centricon Plus-70 Centrifugal Filter *Experiment 3*	100	0.5	4.9	5.0	6.0			
CIMmultus™-QA (8 mL) *Experiment 1*	2000	20	2.9	3.2	6.0	1	1400 €	10 h
CIMmultus™-QA (1 mL) *Experiment 2*	600	8	3.4	3.1	5.1	1	1400 €	8 h
CIMmultus™-SO3 (1 mL) *Experiment 1*	900	8	1.1	2.3	0.5	1	1400 €	8 h
PEG-based concentration *Experiment 1*	35	0.4	2.6	3.4	4.9	5	255 €	5 h
Skimmed Milk-based concentration *Experiment 1*	200	0.8	− 1.7	− 0.4	4.3	8	206 €	8 h
Vivacell 100. 30.000 MWCO PES *Experiment 1*	150	0.5	− 1.0	0.5	2.5	8	248 €	7 h
Wizard Enviro TNA Kit; *Experiment 3*	40	1	6.7	6.9	5.4	14	175 €	7 h

Associated sample volume for load (start) and elution (end), number of samples and time used per batch and price per sample are also shown

**Fig. 2:**
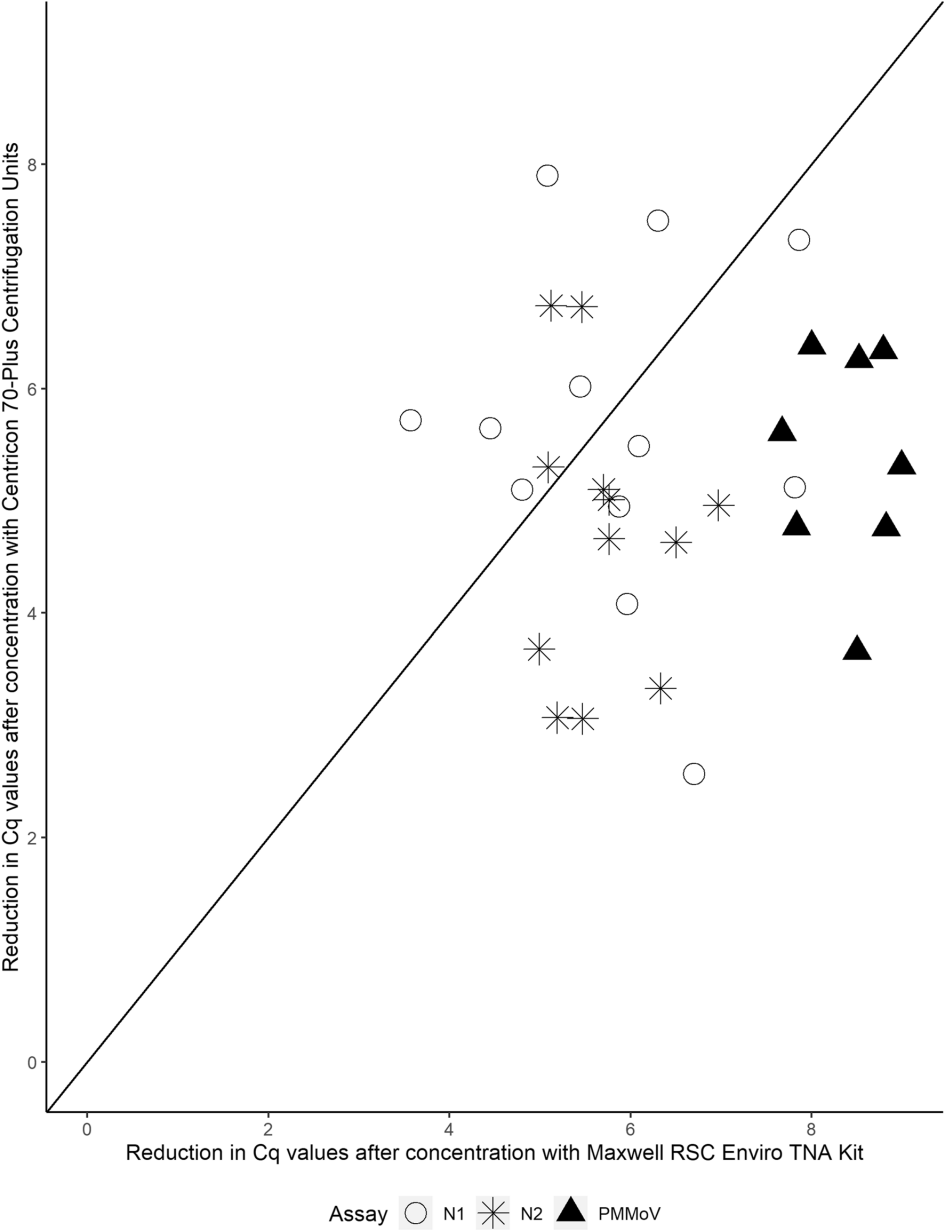
Reduction in average Cq values (*n* = 3) for N1, N2 and PMMoV assays achieved after concentration of real wastewater samples depending on the concentration methods used (Maxwell RSC Enviro TNA Kit or Centricon Plus-70 Centrifugal Filters). Each point represents the relation between the Cq reductions obtained for each target when using the two concentration methods on a given wastewater sample. Points below the line show sample and assay combinations that were concentrated with greater efficiency using Maxwell RSC Enviro TNA Kit and points above the line represent the ones concentrated more efficiently with Centricon Filtration Units and Qiagen extraction step. Figure created in RStudio v.1.2.1106

All three independent experiments, involving three different wastewater samples, Centricon Plus-70 and Wizard Enviro TNA kit, resulted in the lowest Cq values for SARS-CoV-2 assays in the concentrated samples, which would result in the most sensitive RT-qPCR detection of the virus in wastewater. Based on our results these two approaches were further compared using real wastewater samples containing SARS-CoV-2. For this comparison, Wizard Enviro TNA Kit (PROMEGA, USA, A2991) was substituted with Maxwell RSC Enviro TNA Kit (PROMEGA, USA, AS1831) which uses magnetic-based purification of RNA in the second step of the procedure and enables higher throughput. Maxwell RSC Enviro TNA Kit (PROMEGA, USA, AS1831) and Centricon Plus-70 Centrifugation Filters coupled with QIAmp Viral RNA Mini Kit (Qiagen, USA, 52,906) RNA extraction were compared using 12 different SARS-CoV-2 positive wastewater samples. The concentration efficiency for PMMoV was higher using Maxwell RSC Enviro TNA Kit (PROMEGA, USA, AS1831) for all 12 compared samples: Cq reduction of 7 or above, whereas with Centricon-based method this reduction is in the range of 4–6 Cqs (Fig. 2). On the other hand, for concentration efficiency for SARS-CoV-2 (measured by N1 and N2 assays) there is no clear conclusion as to which method performs better based on the observed Cq reductions.

### Screening for the Optimal RT-qPCR Assay/Master Mix Combination

Analysis of the serial dilutions of the thermally inactivated virus showed that N1 and N2 assays with Zen/Iowa Black probes were 10 × more sensitive than E assay (with either BHQ and BBQ650 quenchers) and 100 × more sensitive than RdRp assay (with BBQ650 quencher) assays, achieving also lower variability between the technical replicates (Table 4). Based on these results subsequent evaluations were done only using N1 and N2 assays.

**Table 4:** Cq values obtained from the dilution series of thermally inactivated viruses in wastewater, tested with different assays and different probe quencher modifications (detailed primer/probe sets and their sequences are presented in Supplementary Information; Table S5)

Dilution Factor*	E (FAM/BHQ)	Variance coefficient	E (FAM/BBQ650)	Variance coefficient	RdRp (FAM/BBQ650)	Variance coefficient	N1 (FAM/ZEN/IOWA)	Variance coefficient	N2 (FAM/ZEN/IOWA)	Variance coefficient
0	25.2		24.0		25.9		23.8		24.7	
	24.6	0.09	24.0	0.00	25.7	0.01	23.6	0.02	24.4	0.03
	24.8		23.9		25.7		23.5		24.4	
10	27.5		27.9		28.8		26.9		27.9	
	27.6	0.01	27.5	0.07	28.9	n/a	26.8	0.00	27.8	0.02
	27.7		27.4		Undetected		26.9		27.6	
10^2^	31.1		39.9		Undetected		30.3		31.2	
	Undetected	n/a	33.8	n/a	Undetected	n/a	30.0	0.16	30.7	0.08
	32.1		31.6		Undetected		29.5		30.7	
10^3^	Undetected		Undetected		Undetected		33.0		34.1	
	Undetected	n/a	Undetected	n/a	Undetected	n/a	33.8	0.16	33.5	0.09
	41.3		Undetected		Undetected		33.3		33.8	
5 × 10^3^	Undetected		Undetected		Undetected		Undetected		36.4	
	Undetected	n/a	Undetected	n/a	Undetected	n/a	Undetected	n/a	Undetected	n/a
	Undetected		Undetected		Undetected		34.4		Undetected	
2.5 × 10^4^	Undetected		Undetected		Undetected		35.6		Undetected	
	Undetected	n/a	Undetected	n/a	Undetected	n/a	Undetected	n/a	Undetected	n/a
	Undetected		Undetected		Undetected		Undetected		36.5	
**1.25 × 10^5^	Undetected	n/a	Undetected	n/a	Undetected	n/a	Undetected	n/a	Undetected	n/a
**6.25 × 10^5^	Undetected	n/a	Undetected	n/a	Undetected	n/a	Undetected	n/a	Undetected	n/a

*Concentration range of undiluted thermally inactivated virus as reported by the manufacturer 4–11 × 109 copies/mL

**all 3 technical replicates were undetected

n/a: not applicable

**Fig. 3:**
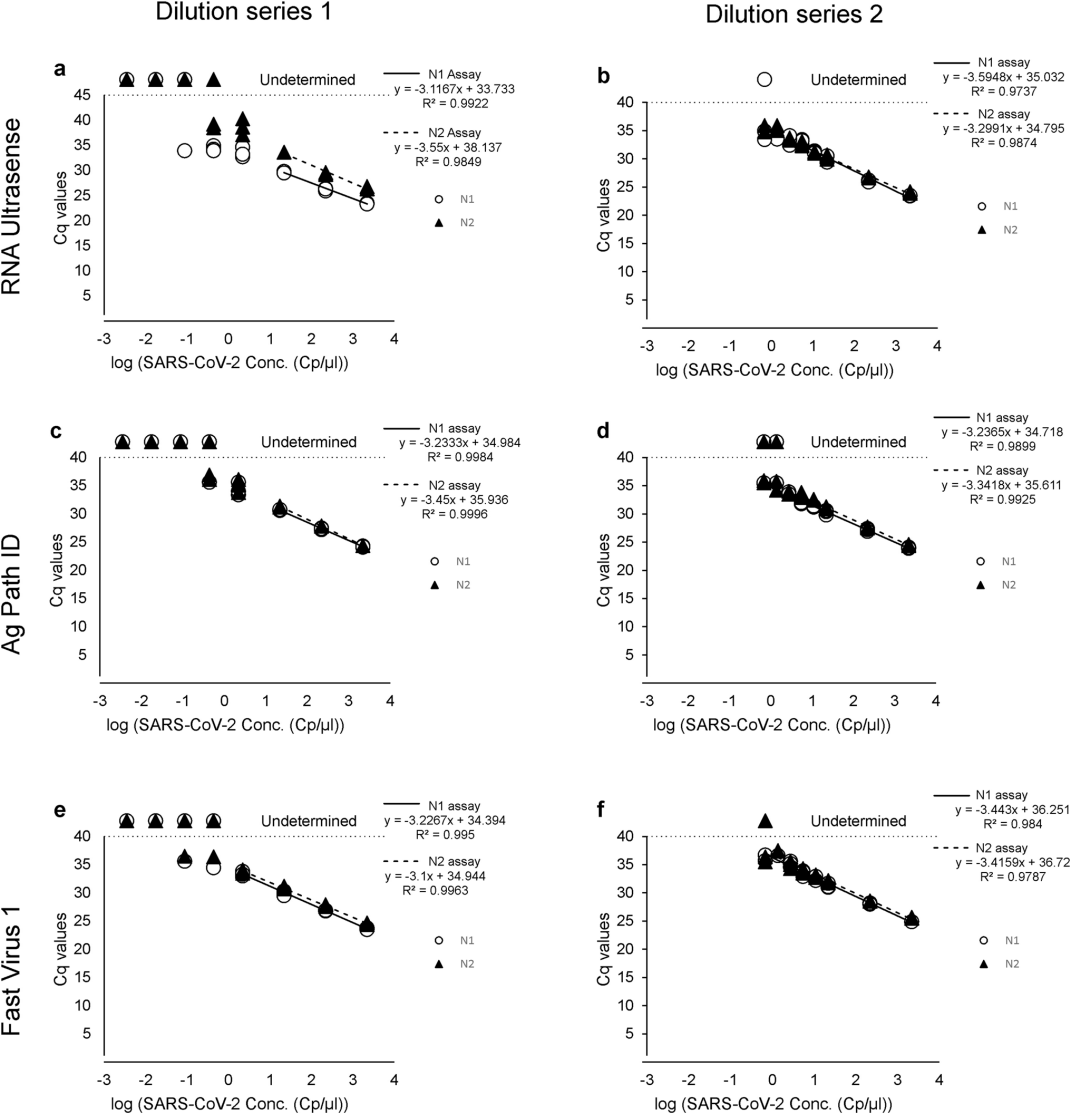
Evaluation of the performance of RT-qPCR (N1 and N2 assays) on serial dilutions 1 (**a**, **c** and **e**) and 2 (**b**, **d** and **f**) of thermally inactivated SARS-CoV-2 in wastewater influent, using three different commercial mastermixes: RNA Ultrasense (**a** and **b**), Ag-Path (**c** and **d**) and Fast Virus (**e** and **f**). Cq values obtained in triplicate measurements for each dilution are plotted against the log of the virus concentration (as calculated from the concentration indicated by the reference material provider). Regression lines were calculated considering only the points that meet the criteria for being included within the quantification range (see Sect. Evaluation of RT-qPCR Assay/Mastermix Combination, and Supplementary Information Table S8 and S9), and obtained regression equation and square error are shown in each graph. The points that gave no signal in the RT-qPCR (undetermined) are shown above the graph for a clearer picture. The Y axis has been moved to the left, as not to overlap with measurements. More detailed information on dilution series, Cq values and other parameters are shown in Supplementary Information Tables S7–10. Figure created in Excel 2016

In a comparison of three different RT-qPCR commercial kits using dilutions of thermally inactivated virus in wastewater with N1 and N2 assays, all 6 assay/kit combinations performed similarly, based on the Cq values obtained for dilutions (Fig. 3). TaqMan Fast Virus 1-Step Master Mix showed lower overall variability between technical replicates, especially at higher dilutions, resulting in larger dynamic range in both dilution series done (Fig. 3, Supplementary Information, Table S8 and Table S9). The slope and intercepts of regression curves, indicative of the amplification efficiency of the RT-qPCR, were most comparable among N1 and N2 assay when using Fast Virus 1-mastermix (Fig. 3). Based on these results we determined for the TaqMan Fast Virus 1-Step Master Mix a practical LoQ for quantification of SARS-CoV-2 in wastewater of 0.69 copies/µL for N1 and 1.37 copies/µL for N2 assay and an LoD for the detection in wastewater of 0.09 copies/µL) for both assays. Other evaluation parameters and regression equations are available in Supplementary Information Table S10. Besides the performance of the mastermix, TaqMan Fast Virus 1-Step Master Mix was the easiest to implement and modify if needed as it is only one reagent that contains the whole mix, whereas the other two are comprised of 2 or more reagents that have to be mixed just before us.

## Discussion

Adopting a WBE approach for monitoring SARS-CoV-2 in wastewater requires an extensive evaluation of each critical step of the process. Nearly three years into the pandemic several institutions, such as the WHO, CDC, USA, and KWR, Netherlands have publicized guides to implementation that list available options for each step in the analysis (WHO., 2022, CDC, USA, 2022; KWR, Netherlands, 2022), Initially, the rapid onset and progression of COVID-19 pandemic forced environmental virologists globally to come up with optimized protocols and method evaluations in reduced time and with diverse limitations such as reagent shortages. Critical phases in the analysis process include: sample collection, sample admission and storage, concentration and nucleic acid extraction, target detection/quantification and data analysis. Here we focused on the core three parts of the procedure from sample admission to virus detection, demonstrating a method set up in a rapid-evolving environment as it was experienced in our laboratory. The outcome of these evaluations was the establishment of a complete analysis workflow that is being currently used for the official national wastewater monitoring in Slovenia.

Sample storage and processing time could have an impact on the final result, due to the possible degradation of the target. This degradation can be driven by various factors, such as time, temperature and the complex composition of wastewater (Markt et al., 2021). Having this in mind, we conducted an experiment that looked into the stability of both extracted RNA from positive controls (EVA-GLOBAL) and thermally inactivated SARS-CoV-2 virus in wastewater at different temperatures for different time frames. We selected the two different spike materials to simulate two extreme forms in which the virus could be present in the sample. Neither of the two reference materials used is likely to entirely resemble the shape in which the virus exists in wastewater and its actual storage stability; however, they are close approximations of two extreme structural viral forms, namely, packed particles versus naked genomic RNA. Based on the obtained results we could see that extracted RNA from positive controls (EVA-GLOBAL) degrades faster in wastewater in comparison to inactivated virus particles. As visible in Fig. 1a, extracted RNA gradually degraded in wastewater over the course of 7 days at + 4 °C, with the biggest change occurring in the first 24 h of storage. When RNA-spiked wastewater was stored frozen at either − 20 °C or − 80 °C, we observed an increase of cca. 2 RT-qPCR Cq values already at 24-h storage. Longer storage times did not translate into higher Cq increase, suggesting that the freeze-thawing cycle required when analysing samples stored at such temperatures could have a larger influence than the storage time itself on the observed increase by affecting the RNA integrity (Fig. 1c and e). Comparable results were also found in wastewater samples with naturally occurring SARS-CoV-2 indicating cca. 3 RT-qPCR Cq values increase in samples that were first frozen on −20 °C compared to ones stored at 4 °C (Qiu et al., 2022). In the case of the inactivated virus spiked in wastewater (Fig. 1b, d and f), we did not observe any increase in the Cq values at none of the temperatures tested, suggesting that the RNA, when protected by the capsid and the lipid envelope is more resilient to environmental degradation than in free RNA form. There is no conclusive information in the literature on the structural form in which SARS-CoV-2 exists in wastewater on the route from households to the WWTP, although suggestions have been made that the virus is present in the form of fully intact enveloped particles (Robinson et al., 2022). However, it can be expected that unlike enteric viruses, which are highly stable in the environment (Sanchez et al., 2016), SARS-CoV-2 enveloped particles will degrade faster in such a milieu, exposing their RNA over time making it susceptible to degradation, as confirmed by our results with RNA from positive controls (EVA-GLOBAL) spiked in wastewater. Since routinely collected samples represent a 24 h-flow-dependent composite sample, the viruses are exposed for an additional 24 h to ambient temperatures during the sampling and in this time, degradation of exposed SARS-CoV-2 RNA can continue.

Based on the available literature early in the pandemic, the first concentration protocol we set up was Centricon Plus-70 Centrifugal Filters coupled with QIAmp RNA Mini Kit for RNA extraction. We checked if the protocol was fit for purpose with two different spike materials in both tap and wastewater and the initial results confirmed the applicability of the protocol, based on the concentration efficacy derived from the reduction of Cq value after the concentration step (Table 1). Additionally, we saw that reference material consisting of synthetic RNA is degraded fast in wastewater, faster and to a higher extent than the RNA from positive controls (EVA-GLOBAL), and thus does not represent a good spike-in for testing the SARS-CoV-2 degradation in real wastewater samples. The rapid fast degradation of both synthetic RNA and RNA from positive controls (EVA-GLOBAL) in wastewater is something to expect considering the complex composition of wastewater influent, which very likely contains substances that cause degradation of RNA. Our results also showed that Centricon Plus-70 concentrated both SARS-CoV-2 and PMMoV similarly. This confirmed the usability of naturally occurring PMMoV as a tool for normalizing the SARS-CoV-2 measurements for changes in faecal load in the wastewater and as concentration efficiency control when using this protocol. The analysis done on 3 replicates from concentration to detection step of a SARS-CoV-2-positive wastewater sample resulted in high repeatability of the concentration using the Centricon protocol, further confirming that this protocol was fit for its purpose (Table 2).

Due to the supply shortages that were experienced globally during the pandemic we decided to test other protocols that could be used instead or alongside Centricon Units. The main parameter to evaluate the concentration step was the measured reduction in Cq values for both SARS-CoV-2 and PMMoV. For all method comparisons we used real wastewater samples. Based on the results we immediately excluded protocols using skimmed milk, Vivacell 100, and CIM-SO3, as they resulted in none or suboptimal concentration (Cq reduction) of SARS-CoV-2. Possible explanations as to why these methods did not work are mainly related to the charge, form and size at which the SARS-CoV-2 virus is present in wastewater. The Vivacell 100 used here, had a molecular cut-off point of 30,000 kDa which is significantly higher than 10,000 kDa in Centricon units. It is also possible that the different material of which the filters are made (polyether sulfone in Vivacell vs cellulose acetate in Centricons) plays a role in the losses due to non-specific binding of viral particles or viral RNA to the filter. Monolithic column CIM-SO3 is negatively charged so its failure likely means that SARS-CoV-2. Skimmed milk flocculation and, especially PEG precipitation are methods that have been widely used in environmental virology for decades (Hamza et al., 2009). Skimmed milk did not concentrate SARS-CoV-2 at all; however, it concentrated PMMoV optimally. PMMoV is known to be present in wastewater as intact infective rod-shaped particles (Bačnik et al., 2020) protected by a protein capsid and thus, can flocculate optimally with the skimmed milk protocol suggesting that SARS-CoV-2 might be present in the wastewater samples in a form difficult to flocculate, likely different to that of intact enveloped particles. It could also explain why another method based on virus particle precipitation, PEG, also concentrated PMMoV better than SARS-CoV-2. It is also worth noting that here we tested just a single variation of both the skimmed milk and PEG protocols forced by the time constraints and requirements of the ongoing SARS-CoV-2 monitoring. Additional optimizations or choosing other variations of the protocols could result in a better performance also for SARS-CoV-2, for example, in the case of PEG precipitation, the initial step of removing solids used here may have resulted in about 30% reduction in recovery (Kaya et al., 2022) and extension of the incubation period to overnight, not used here, could have helped to improve the yield (Farkas et al., 2017). CIM-QA column gave acceptable results, with Cq reductions of approximately 3 for SARS-CoV-2 and 5 for PMMoV, but we excluded it mainly due to the length and complexity of the protocol and difficulties for adaptation to high throughput scenarios such as routine high-scale monitoring, which was the final goal of the method adaptation. Apart from the concentration efficiency of the selected method we also took into account the price per sample, batch size and time to process the batch, as these factors also impact the practicality of wide-scale monitoring. The majority of the methods displayed acceptable prices per sample (apart from column-based methods) and were able to support scaling up. The outlier was the Wizard Enviro TNA Kit from Promega (parameters for Maxwell RSC Enviro TNA Kit are similar but not displayed here) which provided both the lowest price per sample and the highest number of samples per batch. Ultimately, the only two methods that efficiently concentrated both SARS-CoV-2 and PMMoV were Centricon units coupled with Qiagen extraction and either of two tested kit versions from Promega (Wizard Enviro TNA Kit and Maxwell RSC Enviro TNA Kit). These conclusions are in accordance with previous similar studies (Mondal et al., 2021; Pecson et al., 2021), where they also point out that the Promega kits offered higher throughput in comparison to Centricon Filters. The two methods perform similarly for the detection of SARS-CoV-2, but there is a preferentially higher concentration efficiency of PMMoV with the Promega kit (Fig. 2). These findings indicate that although usable, the two methods should they be interchanged, this should be done with caution in cases where PMMoV is used for data normalization. In such cases running both methods in parallel during a transition period will help asses any major changes induced in the data trend by the method exchange.

Two aspects of the RT-qPCR quantification of SARS-CoV-2 were evaluated individually; assay and mastermix selection. As expected, the selected assay has an impact on the sensitivity. RdRp assay showed significantly lower sensitivity compared to E assay and N1 and N2 assays by 10 and 100-fold, likely due to a mismatch in the reverse primer annealing region of the RdRp assay (Nalla et al., 2020). When comparing E assay to N1 and N2 assays, we noticed around a tenfold reduction in sensitivity for E assay. The N1 and N2 assays had double quenched probes, as opposed to the single quenched probes used with E assay. The increased sensitivity of N assays after the addition of a second quencher in the probe has been reported elsewhere (Hirotsu et al., 2020). It is likely that RdRp and E assays would have also benefited from using double quenched probes; however, we did not test for this and our final decision was to use the N1 and N2 assays. Using two assays to confirm the presence of the genetic material of the virus in the sample will aid in decision-making in regards to samples with low virus concentration, which could have been more inconclusive if only one assay was employed. It is important to continuously evaluate the sequences of both assays annealing regions in the new emerging variants that might bear mutations in said regions that could affect their efficiency.

Second tested parameter was the choice of the mastermix used for RT-qPCR. Having several mastermix options seemed like a good contingency plan in response to potential reagent shortages. All observed parameters (regression factor, deviation between technical replicates and sensitivity) were comparable (Supplementary Information Table S10); however, TaqMan Fast Virus 1-step mastermix showed a better performance at low virus concentrations and better correlation between N1 and N2 assays (Fig. 3) and was selected as the main mastermix option.

**Fig. 4:**
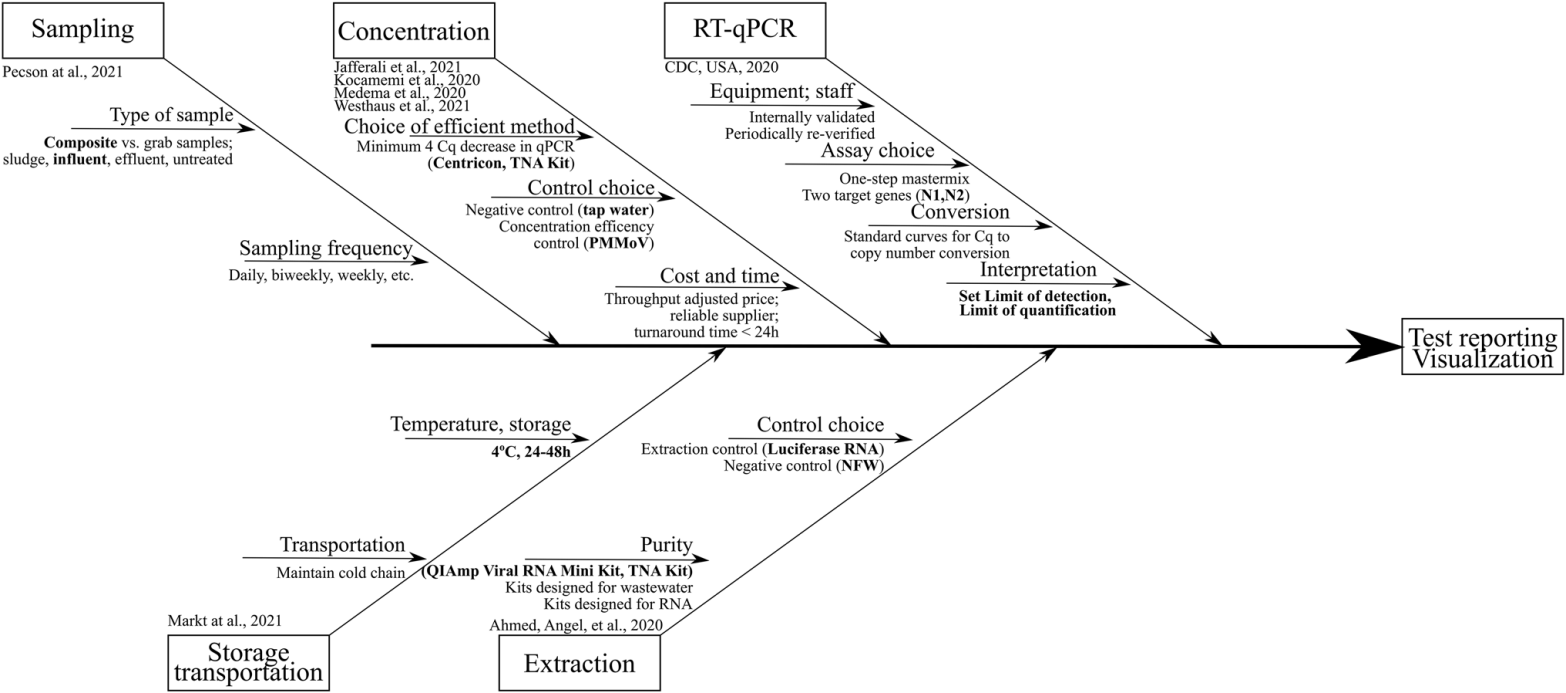
Scheme depicting different factors influencing the outcome of the analysis aimed at detecting SARS-CoV-2 in wastewater. Steps not discussed in this manuscript have appropriate references cited. The steps that were evaluated in the manuscript have the determined choices in bold. The rest of the steps were decided based on the experience of the authors and/or available resources

The outcome of the overall method evaluation described here was the implementation of a complete testing procedure in the work frame of nationwide wastewater monitoring. Taking the time to individually evaluate each step of the procedure allowed us to select the most fit-for-purpose methods. Having properly evaluated methods also enables optimal tech transfer and more accurate inter-laboratory comparisons in the frame of quality assurance environments. Training of additional personnel is also straightforward and time efficient. Thus, we strongly encourage laboratories to implement the WBE approach targeting SARS-CoV-2 or any other viral pathogen, to properly verify each step of the overall procedure. A simplified diagram depicting important factors to be considered in each analysis step is presented in Fig. 4.

## Supplementary Information

Below is the link to the electronic supplementary material.

Supplementary file1 (DOCX 444 kb)

## Data Availability

The datasets generated during and/or analysed during the current study are available from the corresponding author.
