# Supplementary material for: Evaluation of Methods and Processes for Robust Monitoring of SARS-CoV-2 in Wastewater
Source: Food Environ Virol. 2022 Aug 23;14(4):384–400. doi: 10.1007/s12560-022-09533-0 (PMC9398038; doi:10.1007/s12560-022-09533-0)
Supplement: Supplementary file 1 — Supplementary file1 (DOCX 444 kb) [file 12560_2022_9533_MOESM1_ESM.docx]

Supplementary information

Figure 1: Schematic summary of relevant parameters (including starting volume of the sample, elution volume and centrifugation parameters where applicable) for tested concentration methods. From left to right: propylene-glycol (PEG) based and skim milk-based precipitation; ultrafiltration with Centricon Plus-70 Centrifugation units (Centricon, Millipore, Germany, UFC701008) and Vivacell 100 units (Vivacell, Sartorius, Germany, VC1022) describing filter material, molecular weight cut-off (1) and dead stop volumes (2); Direct capture and extraction method using Maxwell RSC Enviro TNA Kit (PROMEGA, USA, AS1831) and monolithic chromatography with columns of different volumes and chemistries (QA – quarternary amine with column volumes of 8 mL and 1 mL; SO3 – sulfonate). RNA extraction was done with either QIAmp Viral RNA Mini Kit (Qiagen, USA, 52906) or Maxwell RSC Enviro TNA Kit (TNA kit, PROMEGA, USA, AS1831).


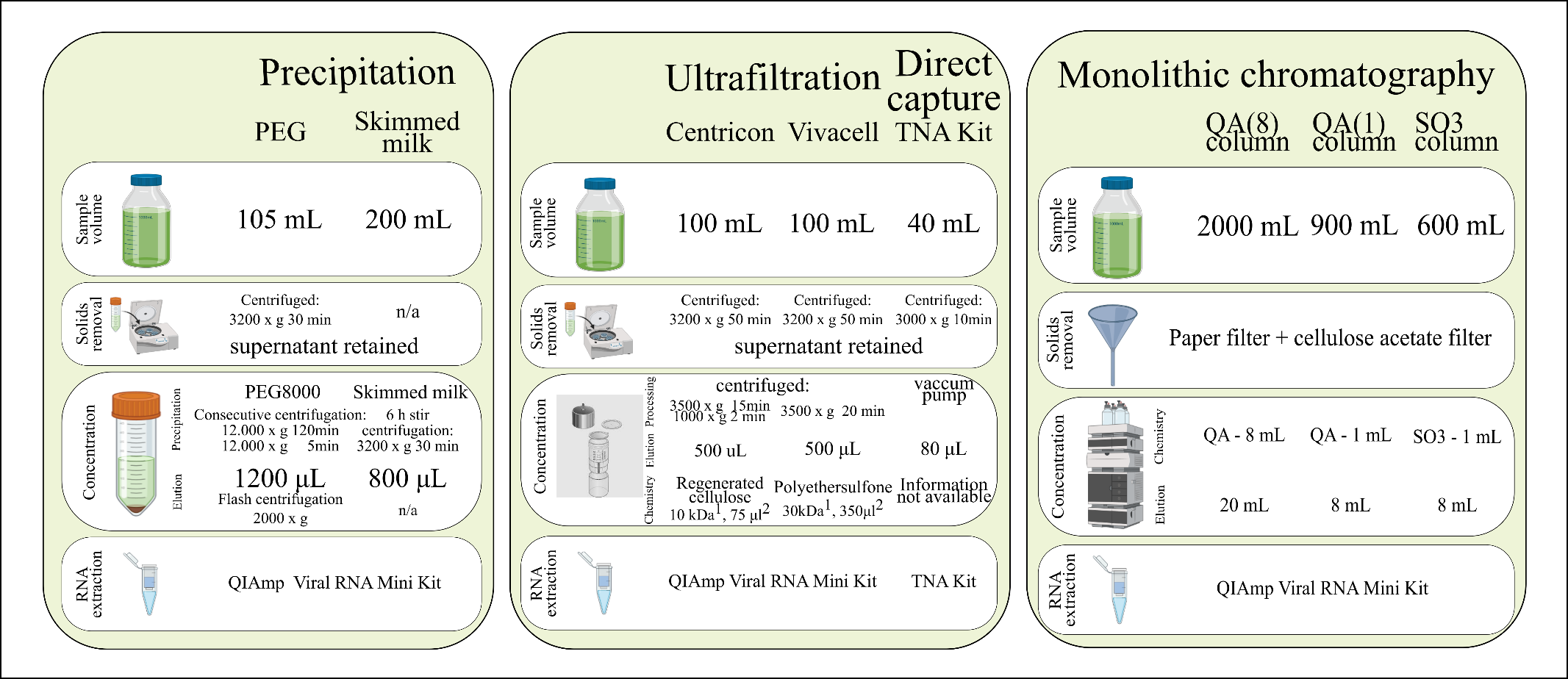


Table 1 Scheme of stability evaluation experiment in wastewater detailing time and temperature at which spikes of inactivated virus and RNA from patients were stored at

| Temperature | +4 ℃ | -20 ℃ | -80 ℃ |
| --- | --- | --- | --- |
| 0 h | RNA control (EVA-GLOBAL); inactivated virus | RNA control (EVA-GLOBAL); inactivated virus | RNA control (EVA-GLOBAL); inactivated virus |
| 24 h | RNA control (EVA-GLOBAL); inactivated virus | RNA control (EVA-GLOBAL); inactivated virus | RNA control (EVA-GLOBAL); inactivated virus |
| 72 h | RNA control (EVA-GLOBAL); inactivated virus | RNA control (EVA-GLOBAL); inactivated virus | RNA control (EVA-GLOBAL); inactivated virus |
| 7 days | RNA control (EVA-GLOBAL); inactivated virus | RNA control (EVA-GLOBAL); inactivated virus | RNA control (EVA-GLOBAL); inactivated virus |

Table 2 Wastewater treatment plants information

| Location | Start of sampling | Population equivalents | Industry wastewater | Household wastewater | Treatment type |
| --- | --- | --- | --- | --- | --- |
| ^1^CWTP Ljubljana | 1.4.2020 | 360.000 | 21 % | 79 % | Mechanical filter, biological filter with nitrification, anaerobic digestion, sludge drying |
| ^1^CWTP Domžale-Kamnik | 31.3.2020 | 149.000 | 10 % | 48 % | Mechanical filter, SBR with tertiary filter, anaerobic digestion, waste processing |
| ^1^CWTP Kranj | 4.8.2020 | 95.000 | 20 % | 70 % | Mechanical filter, cascading biological tertiary filter with active sludge, anaerobic digestion |
| ^1^CWTP Koper | 15.10.2020 | 84.500 | 7 % | 93 % | Mechanical filter, SBR with tertiary filter |
| ^1^CWTP Šaleške doline | 3.4.2020 | Not available | Not available | Not available | Not available |
| ^2^WWTP Celje | 6.4.2020 | 85.000 | 15 % | 85 % | Mechanical filter, tertiary cleaning with aerobic stabilisation of sludge, centrifugal dehydration of sludge |
| ^1^CWTP Maribor | 12.11.2020 | 195.000 | 20 % | 80 % | Mechanical filter, biological filter, sludge processing |

^1^CWTP - Central Wastewater Treatment Plant; ^2^WWTP - Wastewater Treatment Plant

Table 3 Detailed overview of protocol parameters (volume, back pressure limit and type of wash and elution buffers) for different CIM monolithic columns’ chemistries, with the processed and eluted sample volume

| Column chemistry | Column volume | Sample volume | Back Pressure limit | Wash buffer | Elution buffer | Elution volume |
| --- | --- | --- | --- | --- | --- | --- |
| QA (quaternary amine) | 8 mL | 2000 mL | 2 MPa | 50 mM HEPES, pH 7 | 50 mM HEPES, 1 M NaCl, pH 7 | 20 mL |
| QA (quaternary amine) | 1 mL | 900 mL | 1,8 MPa | 50 mM HEPES, pH 7 | 50 mM HEPES, 1 M NaCl, pH 7 | 8 mL |
| SO3 (sulfonate) | 1 mL | 600 mL | 1,8 MPa | 50 mM HEPES, pH 7 | 50 mM HEPES, 1 M NaCl, pH 7 | 8 mL |

Table 4 Overview of samples used for method comparison of different virus concentration methods including sample ID, corresponding WWTP, date of sampling and date of processing and methods used for comparison (in addition to the Centricon Plus-70 Centrifugation units) for each specific sample

| Sample ID | WWTP | Date of sampling | Date of processing | Methods used in addition to Centricon Plus-70 Centrifugation units |
| --- | --- | --- | --- | --- |
| WW318/20 | Domžale-Kamnik | 3.11.2020 | 3-5.11.2020 | ALL |
| WW179/21 | Ljubljana | 06.04.2021 | 06.04.2021 | Maxwell RSC Enviro TNA Kit (PROMEGA, AS1831) |
| WW181/21 | Koper | 06.04.2021 | 06.04.2021 | Maxwell RSC Enviro TNA Kit (PROMEGA, AS1831) |
| WW183/21 | Domžale-Kamnik | 06.04.2021 | 06.04.2021 | Maxwell RSC Enviro TNA Kit (PROMEGA, AS1831) |
| WW185/21 | Ljubljana | 08.04.2021 | 08.04.2021 | Maxwell RSC Enviro TNA Kit (PROMEGA, AS1831) |
| WW187/21 | Ljubljana | 07.04.2021 | 08.04.2021 | Maxwell RSC Enviro TNA Kit (PROMEGA, AS1831) |
| WW189/21 | Celje | 08.04.2021 | 08.04.2021 | Maxwell RSC Enviro TNA Kit (PROMEGA, AS1831) |
| WW191/21 | Maribor | 08.04.2021 | 08.04.2021 | Maxwell RSC Enviro TNA Kit (PROMEGA, AS1831) |
| WW193/21 | Velenje-Šoštanj | 08.04.2021 | 08.04.2021 | Maxwell RSC Enviro TNA Kit (PROMEGA, AS1831) |
| WW195/21 | Ljubljana | 13.04.2021 | 13.04.2021 | Maxwell RSC Enviro TNA Kit (PROMEGA, AS1831) |
| WW197/21 | Domžale-Kamnik | 13.04.2021 | 13.04.2021 | Maxwell RSC Enviro TNA Kit (PROMEGA, AS1831) |
| WW198/21 | Koper | 13.04.2021 | 13.04.2021 | Maxwell RSC Enviro TNA Kit (PROMEGA, AS1831) |
| WW200/21 | Kranj | 13.04.2021 | 13.04.2021 | Maxwell RSC Enviro TNA Kit (PROMEGA, AS1831) |

Table 5 Overview of primer/probe sets and their sequences including number and type of quenchers in different probes used for detection of SARS-CoV-2 in RT-qPCR experiments

| Target gene | Quencher | | Sequence |
| --- | --- | --- | --- |
| E | Black Hole Quencher (BHQ™) | Fw | 5’-ACAGGTACGTTAATAGTTAATAGCGT-3’ |
|  |  | Rw | 5’-ATATTGCAGTACGCACACA-3’ |
|  |  | Probe | 5’-FAM-ACACTAGCCAACCTTACTGCGCTTC-BHQ-3’ |
|  | BlackBerry® Quencher 650 (BBQ-650®) | Fw | 5’-ACAGGTACGTTAATAGTTAATAGCGT-3’ |
|  |  | Rw | 5’-ATATTGCAGTACGCACACA-3’ |
|  |  | Probe | 5’-6-FAM-ACACTAGCCATCCTTACTGCGCTTCG-BBQ-1-3’ |
| RdRp | BlackBerry® Quencher 650 (BBQ-650®) | Fw | 5’-GTGARATGGTCATGTGTGGCGG-3’ |
|  |  | Rw | 5’-CARATGTTAAASACACTATTAGCATA-3’ |
|  |  | Probe | 5’-6-FAM-CAGGTGGAACCTCATCAG?AGATGC-BBQ-1-3’ |
| N1 | ZEN™ and Iowa Black^®^ FQ | Fw | 5’-GACCCCAAAATCAGCGAAAT-3’ |
|  |  | Rw | 5’-TCTGGTTACTGCCAGTTGAATCTG-3’ |
|  |  | Probe | 5’-[FAM]-ACCCCGCAT-[ZEN]-TACGTTTGGTGGACC-[3IABkFQ]-3’ |
| N2 | ZEN™ and Iowa Black^®^ FQ | Fw | 5’-TTACAAACATTGGCCGCAAA-3’ |
|  |  | Rw | 5’-GCGCGACATTCCGAAGAA-3’ |
|  |  | Probe | 5’-[FAM]-ACAATTTGC-[ZEN]-CCCCAGCGCTTCAG-[3IABkF]-3’ |

Table 6 Serial dilution scheme for the thermally inactivated virus in wastewater with associated copy number per µL. Dilutions were applied to evaluate different assays and as dilution series (1) for the evaluation of different mastermixes.

| Dilution factor | Estimated copy number (copies/µL)* |
| --- | --- |
| 0 | 2198.19 |
| 10 | 219.82 |
| 100 | 21.98 |
| 1.000 | 219 |
| 5.000 | 0.439 |
| 25.000 | 0.088 |
| 125.000 | 0.017 |
| 625.000 | 0.003 |

*copy numbers estimate based on provided values by INSTAND.

Table 7 Serial dilution scheme for the thermally inactivated virus in wastewater with associated copy number per µL. Dilution series (2) was applied to evaluate different mastermixes.

| Dilution factor | Estimated copy number (copies/µL)* |
| --- | --- |
| 0 | 2.198.19 |
| 10 | 219.82 |
| 100 | 21.98 |
| 200 | 10.99 |
| 400 | 5.49 |
| 800 | 2.74 |
| 1.600 | 1.37 |
| 3.200 | 0.687 |
| 6.400 | 0.343 |

*copy numbers estimate based on provided values by INSTAND.

Table 8 Obtained Cq values and corresponding variance coefficients between technical replicates for the 3 tested mastermixes and a dilution series (1) of the experiment with a lower resolution of dilutions of used thermally inactivated virus

| Mastermix | | RNA Ultrasense | | | | | AgPath | | | | FastVirus | | | |
| --- | --- | --- | --- | --- | --- | --- | --- | --- | --- | --- | --- | --- | --- | --- |
| Dilution factor / Assay | Concentration (copies/µL) | Cq  N1 | Variance coefficient | Cq  N2 | Variance coefficient | Cq  N1 | | Variance coefficient | Cq  N2 | Variance coefficient | Cq  N1 | Variance coefficient | Cq  N2 | Variance coefficient |
| 0 | 2198 | 23.5 | 0.01 | 26.8 | 0.07 | 24.3 | | 0.01 | 24.4 | 0.00 | 23.8 | 0.0 | 24.7 | 0.03 |
|  | 2198 | 23.5 |  | 26.3 |  | 24.1 | |  | 24.4 |  | 23.6 |  | 24.4 |  |
|  | 2198 | 23.3 |  | 26.4 |  | 24.2 | |  | 24.4 |  | 23.5 |  | 24.4 |  |
| 10 | 219.8 | 25.9 | 0.07 | 29.2 | 0.04 | 27.5 | | 0.02 | 27.8 | 0.00 | 26.9 | 0.00 | 27.9 | 0.02 |
|  | 219.8 | 26.4 |  | 29.3 |  | 27.2 | |  | 27.9 |  | 26.8 |  | 27.8 |  |
|  | 219.8 | 26.3 |  | 29.6 |  | 27.4 | |  | 27.9 |  | 26.9 |  | 27.6 |  |
| 100 | 21.98 | 29.7 | 0.02 | 33.6 | 0 | 30.6 | | 0.01 | 31.3 | 0.01 | 30.3 | 0.16 | 31.2 | 0,08 |
|  | 21.98 | 29.8 |  | 33.6 |  | 30.6 | |  | 31.2 |  | 30.0 |  | 30.7 |  |
|  | 21.98 | 29.5 |  | 33.6 |  | 30.8 | |  | 31.4 |  | 29.5 |  | 30.7 |  |
| 1000 | 2.2 | 32.7 | 0.97 | 40.3 | 2.56 | 33.4 | | 1.29 | 33.8 | 1.33 | 33.0 | 0.1633333 | 34.1 | 0.09 |
|  | 2.2 | 34.6 |  | 38.6 |  | 34.0 | |  | 35.1 |  | 33.8 |  | 33.5 |  |
|  | 2.2 | 33.2 |  | 37.1 |  | 35.6 | |  | 36.1 |  | 33.3 |  | 33.8 |  |
| 5000 | 0.44 | 34.9 | 0.26 | 38.5 | 0.24 | Undetected | | N/A | Undetected | 0.32 | Undetected | N/A | 36.4 | N/A |
|  | 0.44 | 34.2 |  | 39.2 |  | 35.6 | |  | 36.1 |  | Undetected |  | Undetected |  |
|  | 0.44 | 33.9 |  | Undetected |  | Undetected | |  | 36.9 |  | 34.4 |  | Undetected |  |
| 25000 | 0.09 | Undetected | N/A | Undetected | N/A | Undetected | | N/A | Undetected | N/A | 35.6 | N/A | Undetected | N/A |
|  | 0.09 | Undetected |  | Undetected |  | Undetected | |  | Undetected |  | Undetected |  | Undetected |  |
|  | 0.09 | 33.9 |  | Undetected |  | Undetected | |  | Undetected |  | Undetected |  | 36.5 |  |
| 125000* | 0.02 | Undetected | N/A | Undetected | N/A | Undetected | | N/A | Undetected | N/A | Undetected | N/A | Undetected | N/A |
| 625000* | 0.003 | Undetected |  | Undetected |  | Undetected | |  | Undetected |  | Undetected |  | Undetected |  |

* All 3 repetitions on RT-qPCR were determined as Undetected

Table 9 Obtained Cq values and corresponding variance coefficients between technical replicates for the 3 tested mastermixes and dilution series (2) of the experiment with higher resolution of dilutions of used thermally inactivated virus

| Mastermix | | RNA Ultrasense | | | | | AgPath | | | | | FastVirus | | | |
| --- | --- | --- | --- | --- | --- | --- | --- | --- | --- | --- | --- | --- | --- | --- | --- |
| Dilution factor / Assay | Concentration (copies/µL) | Cq  N1 | Variance coefficient | Cq  N2 | Variance coefficient | Cq  N1 | | Variance coefficient | Cq  N2 | Variance coefficient | Cq  N1 | | Variance coefficient | Cq  N2 | Variance coefficient |
| 0 | 2198 | 23.5 | 0.00 | 23.9 | 0.02 | 24.1 | | 0.01 | 24.6 | 0.01 | 24.9 | | 0.00 | 25.7 | 0.01 |
|  | 2198 | 23.4 |  | 24.2 |  | 23.9 | |  | 24.4 |  | 24.9 | |  | 25.6 |  |
|  | 2198 | 23.5 |  | 24.0 |  | 23.9 | |  | 24.4 |  | 24.9 | |  | 25.5 |  |
| 10 | 219.8 | 26.1 | 0.02 | 26.6 | 0.01 | 26.9 | | 0.06 | 27.5 | 0.01 | 28.0 | | 0.01 | 28.6 | 0.00 |
|  | 219.8 | 26.2 |  | 26.8 |  | 27.4 | |  | 27.7 |  | 28.1 | |  | 28.5 |  |
|  | 219.8 | 25.9 |  | 26.6 |  | 27.2 | |  | 27.7 |  | 28.2 | |  | 28.6 |  |
| 100 | 21.98 | 30.5 | 0.40 | 30.7 | 0.17 | 30.6 | | 0.17 | 31.1 | 0.04 | 31.0 | | 0.14 | 31.8 | 0.04 |
|  | 21.98 | 29.4 |  | 30.5 |  | 30.4 | |  | 30.9 |  | 31.7 | |  | 32.2 |  |
|  | 21.98 | 29.4 |  | 29.9 |  | 29.8 | |  | 31.3 |  | 31.1 | |  | 32.1 |  |
| 200 | 10.99 | 31.4 | 0.04 | Outlier | 0.04 | Outlier | | 0.02 | 32.4 | 0.01 | 32.9 | | 0.16 | 32.7 | 0.06 |
|  | 10.99 | 31.2 |  | 31.0 |  | 31.2 | |  | 32.4 |  | 32.2 | |  | 33.0 |  |
|  | 10.99 | 31.0 |  | 31.3 |  | 31.4 | |  | 32.6 |  | 32.9 | |  | 33.2 |  |
| 400 | 5,49 | 33,4 | 0.28 | 32,5 | 0.17 | 31,8 | | 0.01 | 33.8 | 0.22 | 33.9 | | 0.28 | 33.6 | 0.19 |
|  | 5,49 | 32,4 |  | 32,3 |  | 31,9 | |  | 33.1 |  | 32.9 | |  | 33.5 |  |
|  | 5,49 | 33,2 |  | 33,1 |  | 32.0 | |  | 32.9 |  | 33.7 | |  | 34.3 |  |
| 800 | 2.75 | 32.4 | 0.73 | 33.5 | 0.04 | 33.9 | | 0.05 | 34.0 | 0.08 | 35.2 | | 0.25 | 35.6 | 0.42 |
|  | 2.75 | 33.4 |  | 33.7 |  | 33.9 | |  | 33.5 |  | 35.6 | |  | 34.3 |  |
|  | 2.75 | 34.1 |  | 33.3 |  | 33.5 | |  | 34.0 |  | 34.6 | |  | 35.0 |  |
| 1600 | 1.37 | 33.7 | 0.02 | 35.9 | 0.27 | 35.5 | | N/A | 35.8 | 1.28 | Outlier | | 0 | Outlier | 0.00 |
|  | 1.37 | 33.5 |  | 35.0 |  | Undetected | |  | 34.2 |  | 36.6 | |  | 37.4 |  |
|  | 1.37 | Outlier |  | 35.9 |  | Undetected | |  | Undetected |  | 36.6 | |  | 37.5 |  |
| 3200 | 0.69 | 33.4 | 1.12 | 35.9 | 0.40 | Undetected | | N/A | Undetected | 0.08 | 36.7 | | 0.33 | 35.5 | 0.40 |
|  | 0.69 | 34.9 |  | 35.9 |  | 35.5 | |  | 35.9 |  | 36.7 | |  | 36.4 |  |
|  | 0.69 | Undetected |  | 34.8 |  | Undetected | |  | 35.5 |  | 35.7 | |  | Undetected |  |

Table 10 Performance characteristics, like LoD, LoQ and linear regression, of different matermixes with N1 and N2 assays

| Mastermix | Ultrasense | | AgPath | | VirusFast | |
| --- | --- | --- | --- | --- | --- | --- |
| Parameter | N1 | N2 | N1 | N2 | N1 | N2 |
| Square regression value (dilution series 1) | 0.94 | 0.88 | 0.92 | 0.93 | 0.94 | 0.96 |
| Linear regression equation (dilution series 1) | 3.11x+33.73 | 3.55x+38.14 | 3.23x+34.98 | 3.45x+35.93 | 3.23x+34.39 | 3.10x+34.94 |
| Square regression value (dilution series 2) | 0.97 | 0.97 | 0.98 | 0.99 | 0.98 | 0.99 |
| Linear regression equation (dilution series 2) | 3.59x+35.03 | 3.47x+35.02 | 3.15x+34.51 | 3.34x+35.61 | 3.44x+36.23 | 3.23x+36.30 |
| Practical LoD (copies/µL) | 0.1 | 0.4 | 1.4 | 0.4 | 0.1 | 0.1 |
| Practical LoQ (copies/µL) | 5.5 | 0.7 | 2.7 | 2.7 | 0.7 | 1.4 |

Table 11 Minimum Information for Publication of Quantitative Real-Time PCR Experiments performed in this study

| **Item to check** | **Importance** | **Checklist** | **Comments/ where?** |
| --- | --- | --- | --- |
| **Experimental design** | | | |
| Definition of experimental and control groups | E | Yes | Materials and methods |
| Number within each group | E | Yes | Materials and methods |
| **Sample** | | | |
| Description | E | Yes | Materials and methods |
| Volume/mass of sample processed | D | Yes | Materials and methods |
| Microdissection or macrodissection | E | N/A | / |
| Processing procedure | E | Yes | Materials and methods |
| If frozen - how and how quickly? | E | N/A | / |
| If fixed - with what, how quickly? | E | N/A | / |
| Sample storage conditions and duration (especially for FFPE samples) | E | Yes | Fridge, +4°C, up to 48h |
| **Nucleic acid extraction** | | | |
| Procedure and/or instrumentation | E | Yes | Materials and methods |
| Name of kit and details of any modifications | E | Yes | Materials and methods |
| Details of DNase or RNAse treatment | E | N/A | / |
| Contamination assessment (DNA or RNA) | E | Yes | Negative control of isolation |
| Nucleic acid quantification | E | N/A |  |
| Instrument and method | E | N/A | / |
| Purity (A260/A280) | D | N/A | / |
| Yield | D | N/A | / |
| RNA integrity method/instrument | E | N/A | / |
| RIN/RQI or Cq of 3' and 5' transcripts | E | N/A |  |
| Inhibition testing (Cq dilutions, spike or other) | E | Yes | Cq dilutions |
| **Reverse transcription** | | | |
| Complete reaction conditions | E | Yes | Materials and methods |
| Amount of RNA and reaction volume | E | Yes | Materials and methods |
| Priming oligonucleotide and concentration | E | Yes | Materials and methods |
|  |  |  | Supplementary Table 5 |
| Reverse transcriptase and concentration | E | Yes | TaqMan™ Fast Virus 1-Step Master Mix – M-MLV; AgPath-ID™ One-Step RT-PCR Reagents - ArrayScript™; RNA UltraSense™ One-Step Quantitative RT-PCR System – not available |
| Temperature and time | E | Yes | Materials and methods |
| Manufacturer of reagents and catalogue numbers | D | Yes | Materials and methods |
| Cqs with and without RT | D | No |  |
| Storage conditions of cDNA | D | N/A | / |
| **qPCR target information** | | | |
| If multiplex, efficiency and LOD of each assay. | E | N/A | / |
| Sequence accession number | E | Yes | Material and Methods, cited in respective references |
| Location of amplicon | D | Yes | Material and Methods, cited in respective references |
| Amplicon length | E | Yes | Material and Methods, cited in respective references |
| *In silico* specificity screen (BLAST, etc.) | E | NO |  |
| Sequence alignment | D | NO |  |
| Secondary structure analysis of amplicon | D | N/A | Used validated assay |
| **qPCR oligonucleotides** | | | |
| Primer and probe sequences | E | Yes | Supplementary Table 5 |
| Location and identity of any modifications | E | N/A |  |
| Manufacturer of oligonucleotides | D | Yes | IDT |
| Purification method | D | Yes | HPLC |
| **qPCR protocol** | | | |
| Complete reaction conditions | E | Yes | Materials and methods |
| Reaction volume and amount of cDNA/DNA | E | Yes | Materials and methods |
| Primer, (probe), Mg++ and dNTP concentrations | E | Yes | Material and methods; Manufacture proprietary |
| Polymerase identity and concentration | E | Yes | TaqMan™ Fast Virus 1-Step Master Mix - AmpliTaq™ Fast DNA Polymerase; AgPath-ID™ One-Step RT-PCR Reagents - AmpliTaq Gold DNA Polymerase; RNA UltraSense™ One-Step Quantitative RT-PCR System - DNA Polymerase |
| Buffer/kit identity and manufacturer | E | Yes | Materials and methods |
| Exact chemical constitution of the buffer | D | No | Manufacturer proprietary |
| Additives (SYBR Green I, DMSO, etc.) | E | No | Manufacturer proprietary |
| Complete thermo-cycling parameters | E | Yes | Material and methods |
| Reaction setup (manual/robotic) | D | Yes | Manual set-up |
| Manufacturer of qPCR instrument | E | Yes | Materials and methods |
| **qPCR validation** | | | |
| Evidence of optimization (from gradients) | D | No |  |
| Specificity (gel, sequence, melt, or digest) | E | No | / |
| Standard curves with slope and y-intercept | E | Yes | Figure 3, Supplementary table 10 |
| PCR efficiency calculated from the slope | E | Yes | Figure 3, Supplementary table 10 |
| Confidence interval for PCR efficiency or standard error | D | No | / |
| r2 of the standard curve | E | Yes | Supplementary table 10 |
| Linear dynamic range | E | Yes | Figure 3, Results |
| Cq variation at the lower limit | E | Yes | Figure 3, Supplementary table 8 and 9 |
| Confidence intervals throughout the range | D | No | / |
| Evidence for a limit of detection | E | Yes | Materials and methods; Figure 3, Supplementary table 10 |
| **Data analysis** | | | |
| qPCR analysis program (source, version) | E | Yes | Materials and methods |
| Cq method determination | E | Yes | Materials and methods |
| Outlier identification and disposition | E | N/A | / |
| Results of NTCs | E | Yes | Negative in all cases |
| Justification of number and choice of reference genes | E | Yes | Materials and methods; discussion |
| Description of normalization method | E | N/A |  |
| Number and stage (RT or qPCR) of technical replicates | E | Yes | Materials and methods |
| Repeatability (intra-assay variation) | E | Yes | Results, Supplementary table 8 and 9 |
| Reproducibility (inter-assay variation, %CV) | D | No |  |
| Statistical methods for result significance | E | N/A |  |
| Software (source, version) | E | Yes | Materials and methods |
| Cq or raw data submission using RDML | D | No |  |

E: Essential information, D: Desirable information, N/A: Not applicable
